# Resection and Reconstruction of the Inferior Vena Cava Using a Tunneled Peritoneal Graft for Renal Tumor With Thrombus: Venous Compensation via the Azygos Vein Following Early Graft Occlusion

**DOI:** 10.7759/cureus.76533

**Published:** 2024-12-28

**Authors:** Marcos Andrés Sánchez-Rendon, Marco Alberto Ocaña-Munguia, Gerardo Llamas-Linares, Néstor Méndez-Huerta, José Gustavo Arrambide-Gutierrez, Francisco Vasquez-Fernandez

**Affiliations:** 1 Urology, Hospital Universitario Dr. José Eleuterio González, Universidad Autónoma De Nuevo León, Monterrey, MEX; 2 Radiology, Hospital Universitario Dr. José Eleuterio González, Universidad Autónoma De Nuevo León, Monterrey, MEX; 3 General Surgery, Hospital Universitario Dr. José Eleuterio González, Universidad Autónoma De Nuevo León, Monterrey, MEX; 4 Surgery, Hospital Universitario Dr. José Eleuterio González, Universidad Autónoma De Nuevo León, Monterrey, MEX

**Keywords:** autologous peritoneal graft, collateral venous circulation, inferior vena cava reconstruction, renal cell carcinoma with tumor thrombus, vascular surgery in malignancy

## Abstract

Inferior vena cava (IVC) invasion by tumor thrombus poses a significant surgical challenge, often requiring vascular reconstruction. Standard methods, including prosthetic and autologous vein grafts, have limitations such as infection risks, anticoagulation demands, and increased costs. We present the case of a 66-year-old male with a right renal tumor (T3bN0M0, Neves Zincke II) and gross hematuria, who underwent radical nephrectomy with open thrombectomy. During surgery, extensive IVC invasion was identified, and a 12×7 cm autologous peritoneal graft was used for IVC reconstruction in the absence of other graft options. Postoperative imaging revealed initial patency; however, near-total graft occlusion was observed by day 15, with asymptomatic compensation via the azygos vein. Pathology revealed clear cell renal cell carcinoma with sarcomatoid and rhabdoid features (ISUP/WHO grade 4) and negative surgical margins, and adjuvant pembrolizumab was initiated. This case highlights the utility of autologous peritoneal grafts as an emergency solution when conventional options are unavailable; however, this treatment also carries potential complications. Further research is needed to optimize graft durability and improve long-term outcomes in vascular reconstructions involving the IVC.

## Introduction

Inferior vena cava (IVC) invasion by tumor thrombus represents a significant surgical challenge, particularly when resection requires vascular reconstruction. The standard approaches, including prosthetic grafts, autologous vein grafts, and resection without reconstruction, have well-documented advantages and limitations. For instance, prosthetic materials like polytetrafluoroethylene (PTFE) and Dacron offer durability but carry increased risks of infection and thrombosis, necessitating prolonged anticoagulation [1,2]. Meanwhile, autologous vein grafts, though biocompatible, demand additional surgical incisions and may not always be feasible in patients with venous insufficiency [3,4].

Biological grafts, such as those derived from cadaveric or autologous tissue, have emerged as alternatives with favorable outcomes, particularly in cancer surgery requiring major vascular reconstruction [5,6]. Autologous peritoneal grafts, in particular, are readily available, cost-effective, and do not necessitate prolonged anticoagulation. However, their long-term durability in high-flow vascular systems remains a challenge [6,7].

## Case presentation

Methods

**Figure 1:**
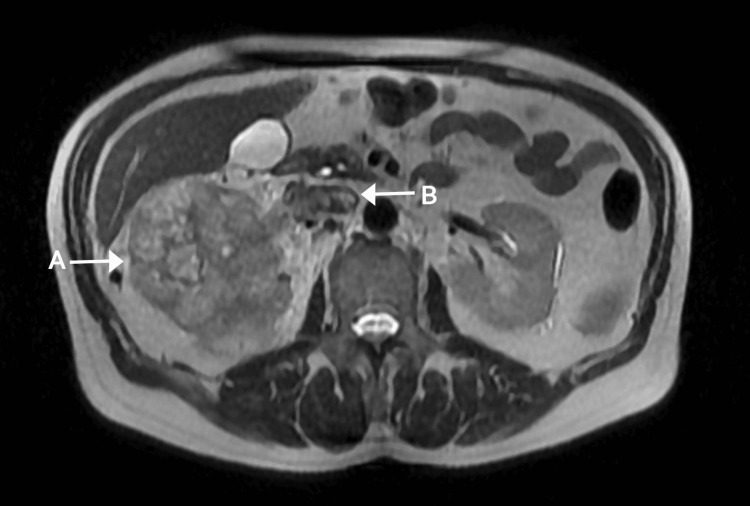
Axial section of angiographic MRI. Arrows: A: renal tumor, B: tumor thrombus.

A 66-year-old male presented with gross hematuria for four months. His medical history included a first-degree atrioventricular block with a preserved left ventricular ejection fraction of 65%. Imaging revealed a 10 cm right renal tumor with a 1.6 cm tumor thrombus extending into the IVC, staged as T3bN0M0 (Neves Zincke II) (Figure 1). Preoperative renal function was preserved, serum creatinine was 1.1 mg/dL (reference value: 0.7-1.3 mg/dL), and the ECOG performance status was 1.

**Figure 2:**
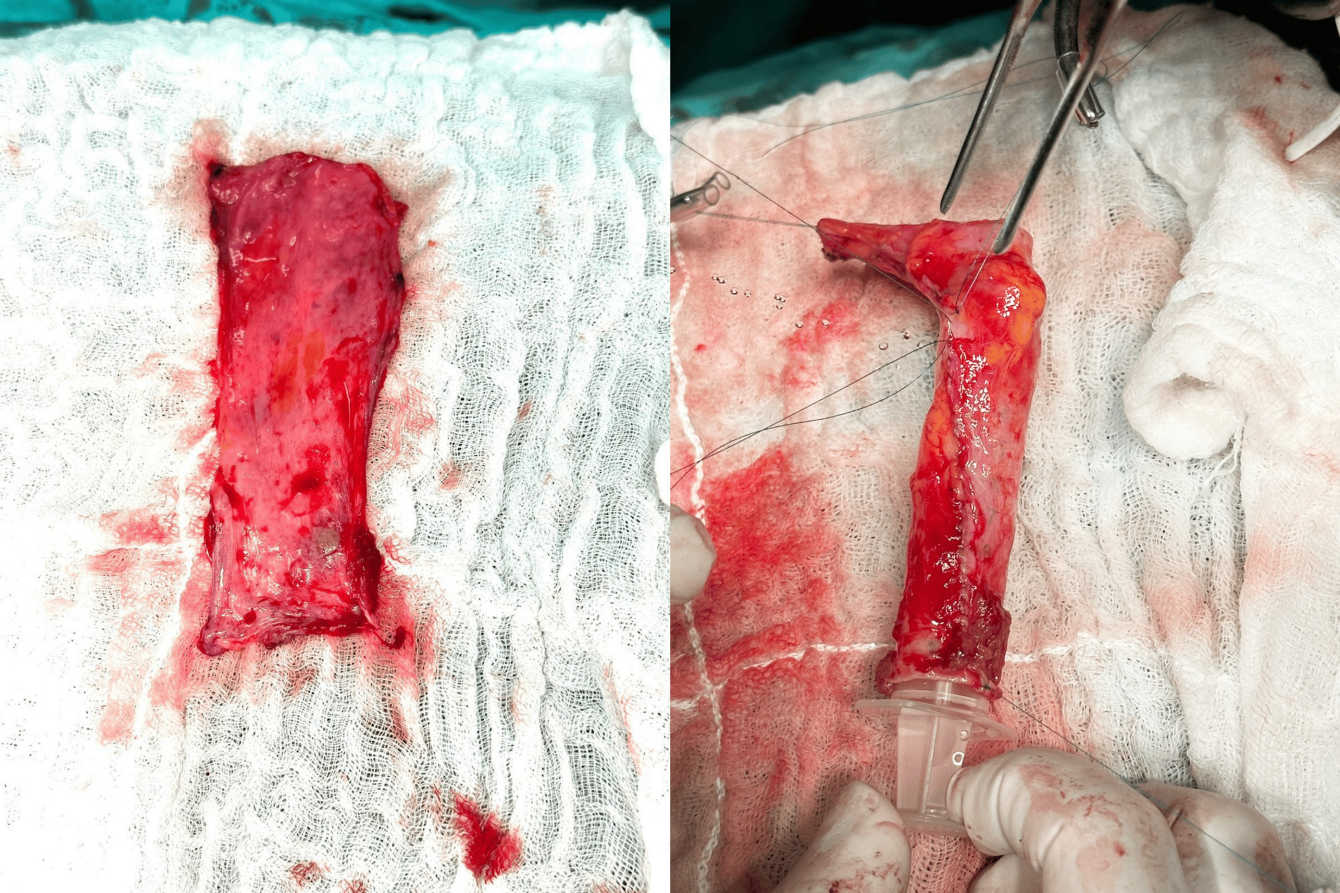
Peritoneal graft and tunneling. In the first figure, we see the rectangular peritoneal graft, which is subsequently tubularized on itself.

**Figure 3:**
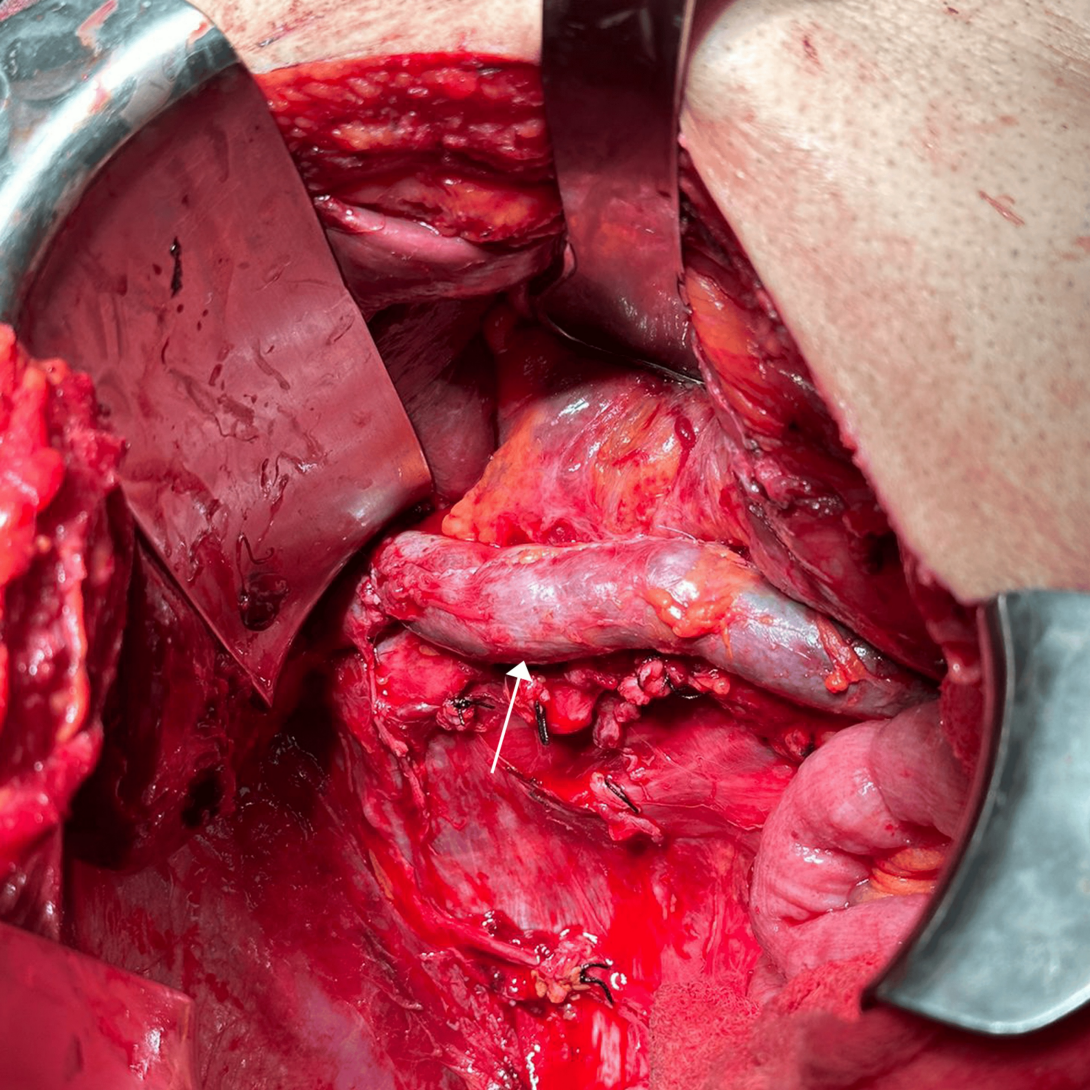
Arrow points to the end-to-end anastomosis of the peritoneal graft.

Radical nephrectomy with open thrombectomy and IVC reconstruction was planned. During surgery, an extensive IVC invasion involving approximately 12 cm was identified. Due to the unavailability of prosthetic or cadaveric grafts, an autologous peritoneal graft was harvested from the lateral abdominal wall (Figure 2). A segment of 15x10 cm of peritoneum was harvested and tubularized, creating a tubular graft with a running 6-0 prolene. Cava vein replacement was successfully performed using a 12 cm tubular graft, with proximal and distal anastomoses, and reinsertion of the contralateral renal vein using running 6-0 prolene (Figure 3).

Results

**Figure 4:**
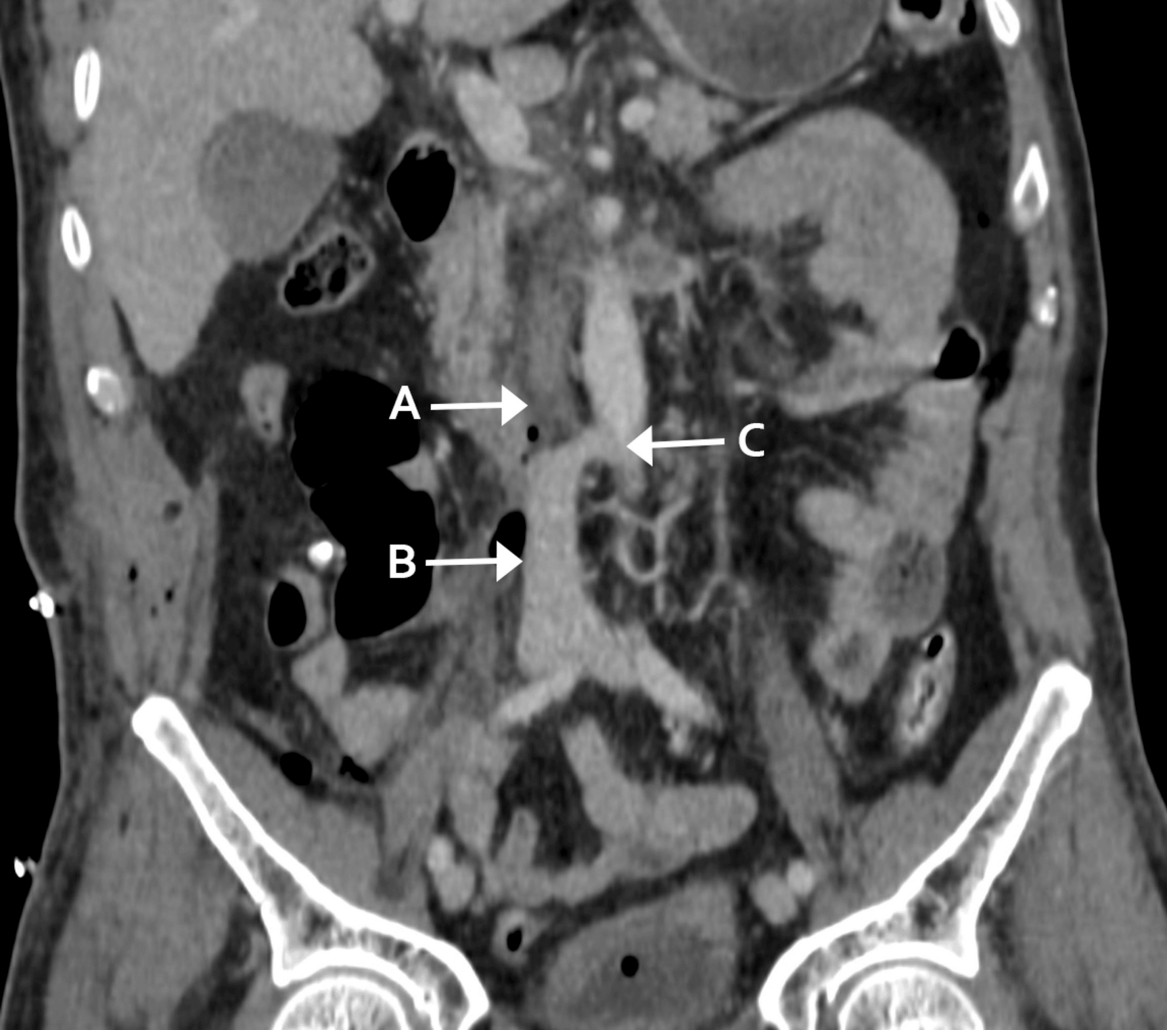
Contrast-enhanced abdominal CT, coronal section. Arrows: A: IVC occlusion; B: IVC; C: azygos vein IVC, inferior vena cava

The patient was admitted to the intensive care unit postoperatively. Low-molecular-weight heparin was initiated six hours after surgery for thromboprophylaxis at a dose of 40 mg subcutaneously every 24 hours. Serum creatinine rose transiently to 1.7 mg/dL but stabilized at 1.4 mg/dL by postoperative day 15. A CT angiogram at 24 hours confirmed graft patency with minor fibrin deposits, but a follow-up CT on day 15 revealed near-total occlusion. Additional evaluations included a lower limb Doppler ultrasound, which showed no evidence of deep vein thrombosis, and a transthoracic echocardiogram, which revealed no hemodynamic changes. The patient remained asymptomatic, with azygos vein collateral circulation compensating for venous return (Figure 4).

Histopathological analysis confirmed clear cell renal cell carcinoma with sarcomatoid and rhabdoid features (ISUP/WHO grade 4), with negative surgical margins. Pembrolizumab was initiated as adjuvant therapy.

## Discussion

Comparative graft analysis

Prosthetic materials, such as PTFE and Dacron, provide structural integrity and high-pressure resistance but are associated with increased risks of infection and thrombosis [1,2,4]. Autologous vein grafts, while biocompatible, pose challenges such as donor site morbidity [3,5]. Cadaveric grafts, though anatomically favorable, are often limited by availability and preservation challenges [6].

The use of autologous peritoneal grafts for IVC reconstruction, as described by Coubeau et al., demonstrated favorable outcomes in four patients undergoing multiorgan resections for malignancies involving the retro-hepatic IVC. Their technique, similar to ours, utilized autologous peritoneum due to the unavailability or infeasibility of prosthetic grafts. Notably, Coubeau et al. reported graft patency at four months postoperatively with no major complications such as thrombosis or occlusion [6]. In contrast, our case experienced early graft occlusion by postoperative day 15, despite adequate initial patency and successful collateral circulation via the azygos vein. This difference highlights the variability in outcomes, which may be influenced by factors such as graft handling, venous pressure dynamics, or postoperative management. The success observed by Coubeau et al. reinforces the feasibility of this technique in selected cases, particularly when synthetic grafts are contraindicated. However, our findings underscore the need for further studies to optimize graft preparation, surgical technique, and postoperative anticoagulation protocols to improve long-term outcomes in high-pressure vascular systems like the IVC [6,7].

Role of collateral circulation

Collateral venous networks, such as the azygos system, are vital in maintaining hemodynamic stability in cases of IVC occlusion. Literature indicates that pre-existing collateral pathways significantly reduce the risk of venous congestion, as observed in this case [8,9].

Adjuvant therapy

Pembrolizumab is increasingly recognized as a cornerstone of systemic therapy for high-risk renal cell carcinoma, particularly in tumors with sarcomatoid features [10,11].

## Conclusions

This case illustrates the utility of autologous peritoneal grafts in emergency IVC reconstruction when conventional grafts are unavailable. However, their limitations in high-pressure vascular systems underscore the possible complications. Collateral circulation, particularly via the azygos system, plays a pivotal role in compensating for graft failure. Pembrolizumab is integral to systemic therapy for aggressive renal cell carcinoma.
